# Introducing the Immunovirology Section of *Journal of Translational Medicine*

**DOI:** 10.1186/1479-5876-8-3

**Published:** 2010-01-14

**Authors:** Luigi Buonaguro

**Affiliations:** 1 Lab of Molecular Biology and Viral Oncogenesis & AIDS Reference Center, Istituto Nazionale Tumori "Fond G Pascale", Naples, Italy and Institute of Human Virology, University of Maryland School of Medicine, Baltimore, MD, USA

## Abstract

A new section of the *Journal of Translational Medicine *has been launched, devoted to promoting the field of Immunovirology.

## 

The general purpose of translational research is to transfer in humans novel therapeutic strategies developed through experimentation which, itself, should be finely tuned by clinical observation. The approach, indeed, should be applied in a "two-way road" fashion: Bench to Bedside and Bedside to Bench.

In this general concept, the study of host-pathogen interrelation and of immunological preventive as well as therapeutic approaches needs to be conducted in a "two-way road" fashion. In this regard, in addition to classic immunological and virological evaluations which will always provide invaluable information, an increasing large number of technologies are available to address specific questions related to the host-pathogen "clash" within a system-level scenario.

The systems biology approach, indeed, represents the comprehensive and quantitative analysis of the interactions between components of biological systems over time. It enables wide-ranging analysis of the complex interactions that maintain the balance between natural as well as vaccine-induced host defence and inflammatory disease. Moreover, it provides essential information on the susceptibility and adaptation of the pathogens to the host-defence strategies. In this context, the design of new preventive as well as therapeutic immunotherapy or vaccine strategies will be increasingly driven by a knowledge-based methodology, ending the empirical approach which has mostly characterized the field.

The Immunovirology section of *Journal of Translational Medicine *within the overall aims of JTM, will be mainly focused on translation research data related to virology and immunity to virus-based diseases, with a particular interest in human oncogenic viruses. Studies focused on the role of innate as well as adaptive immunity in the establishment, containment and/or progression of viral infections up to cancer, including vaccine and adjuvanting strategies, will be considered for publication. Classic immunology as well as more recent, less established "omics" approaches (genomics, proteomics, immunogenomics, pharmacogenomics) will be hosted in the Immunovirology section of JTM, with the scope of their validation in a "bench-to-clinic" translational perspective.

In the spirit of *Journal of Translational Medicine*, including rapid publication, open-access, high standard peer review process, the Immunovirology section provides a venue for publication of original research articles, literature reviews, opinion/position articles, meeting reports, monographic issues and a forum to discuss the hot issues in Immunology and Virology. The section is proud to include members of high scientific reputation in its Board who will ensure the high standard level. On behalf of the Editorial Board, we invite you to submit your scientific contributions, giving the opportunity to the Immunovirology section to successfully contribute to the advancement of the field.

Luigi Buonaguro, Section Editor Immunovirology

